# ETEC biofilms are regulated by magnesium and lactate bioavailability

**DOI:** 10.1128/iai.00243-25

**Published:** 2025-08-11

**Authors:** Ian E. Hollifield, Kristen L. Clement, Kaylynn A. Fernando, Michelle D. Blythe, Jacob P. Bitoun

**Affiliations:** 1 Department of Microbiology and Immunology, Tulane University School of Medicine 12255, New Orleans, Louisiana, USA; St. Jude Children's Research Hospital, Memphis, Tennessee, USA

**Keywords:** *Escherichia* toxins, biofilms

## Abstract

The prevailing dogma is that enterotoxigenic *Escherichia coli* (ETEC) use plasmid-borne colonization factors (CFs) to adhere to the intestinal epithelium, where the organisms proliferate and produce their diarrhea-causing virulence factors, the heat-stable (ST) and/or heat-labile (LT) enterotoxins. However, vaccines that target major CF antigens fail to induce complete protective immunity, indicating that ETEC may also use other antigens to colonize the small intestines. We previously demonstrated that ST intoxication limits magnesium bioavailability in the intestinal lumen, but the role of magnesium in ETEC pathogenesis has not been rigorously evaluated. Here, we demonstrate that addition of magnesium at concentrations found in the intestinal mucosa promotes biofilm formation in ETEC H10407 and other clinical isolates, especially in the presence of lactate. ETEC H10407 biofilms fail to express high levels of colonization factor antigen I(CFA/I) fimbriae, but ETEC H10407 biofilms remain significantly better than planktonic counterparts at adhering to intestinal epithelial cells. Furthermore, ETEC H10407 biofilms are more acid-resistant than their planktonic counterparts, indicating that biofilms may promote survival through gastric acidity. Finally, using intragastric infection of neonatal mice, ETEC H10407 biofilms are significantly more virulent than their planktonic counterparts. Scanning electron micrographs of biofilm-infected mice show ETEC H10407 adheres to small intestinal villi. Thus, ETEC may respond to changes in environmental conditions to alter adherence mechanisms. Therefore, identification of biofilm antigens should be prioritized in ETEC vaccine development.

## INTRODUCTION

Enterotoxigenic *Escherichia coli* (ETEC) are common diarrheal pathogens of children and adults in low-to-middle income countries (LMICs) (1). ETEC cause substantial mortality in children under 5 years of age, and children who experience non-fatal episodes of severe diarrheal disease can suffer from other negative health outcomes including growth stunting (2) and cognitive developmental delays (3, 4). The clinical approach to secretory diarrheal disease management focuses on oral rehydration and antibiotic therapies, and there are no licensed vaccines available. Consequently, there is an urgent need to perform mechanistic studies into ETEC pathogenesis to define antigens that can be used to develop vaccines that protect both children and adults from the diarrheal and non-diarrheal burdens caused by these significant enteric pathogens.

Vaccine-mediated immunity should be feasible since children living in LMICs acquire natural immunity to ETEC after natural infection (5), and human challenge studies demonstrate that protective immunity to ETEC develops after infection (6–8). In the classic paradigm, ETEC adhere to the small intestinal epithelium via fimbrial, fibrillar, or afimbrial adhesins called colonization factors (CFs), proliferate, and elaborate the heat-stable (ST) and heat-labile (LT) enterotoxins. Antibodies, particularly secretory IgA, that neutralize toxins and block adhesin binding in the small bowel, protect against ETEC and other enteric infections (9, 10). Therefore, the development of antigens that induce anti-toxin and anti-CF antibodies in the mucosa has been prioritized for ETEC vaccines.

CFs were first identified as critical virulence factors in the 1970s, when a highly diarrheagenic isolate called ETEC H10407 lost a plasmid encoding fimbrial genes and was found to be avirulent in human volunteers (avirulent strain is called ETEC H10407p) (11). Vaccine antigens have been developed that cover major CF types including CFA/I (12), CS3 (13), and CS6 (14). However, landmark epidemiological studies, the Global Enterics Multicenter Study (GEMS) (15) and the Malnutrition and Enteric Disease Study (MAL-ED) (1), have shown that over 30 antigenically different CFs are encoded in the ETEC pan-genome (16). This has complicated deciding which CF antigens should be included in ETEC vaccines. Furthermore, more than half of all sequenced ETEC isolates lack major CFs (1, 17), so advanced development of vaccines targeting non-canonical adhesins has received increased interest. While Ag43 (18), EtpA (19), and TibA (20) are some of these noncanonical antigens, TibA is expressed in a small number of ETEC strains (21), and Ag43 is not unique to the ETEC pathovar (22). Therefore, the identification of novel mechanisms through which ETEC adhere to the intestinal epithelium may be required to decrease the burden of this significant enteric pathogen.

ETEC are defined by the presence of the LT and/or ST enterotoxins, and while immunity to LT can be induced (7), it remains insufficient in the absence of an immunogen that induces protective anti-ST immunity. One of the major technical hurdles in developing anti-ST immunity is overcoming its small (~2 kDa) size. Enterotoxin activities cause large physiological changes in the gut. LT induces increased intracellular cAMP production, while ST induces increased intracellular cGMP production (23, 24). These cyclic nucleotide second messengers then alter the activity of ion transporters (e.g., cystic fibrosis transmembrane regulator and sodium hydrogen exchanger 3) that ultimately increase concentration of chloride and sodium ions in the intestinal lumen, leading to water influx via osmosis (25). Robust secretory diarrhea characteristic of clinical ETEC and cholera infections occurs if the secretory fluid cannot be reabsorbed before it exits the colon (26).

It is largely believed that the enterotoxins provide ETEC with increased dissemination, but LT provides ETEC with a colonization advantage, as seen in both murine and porcine models (27, 28). Because LT and cholera toxin (CT) are ~90% identical in protein structure (29–31), ETEC infections are often compared to *Vibrio cholerae* infections. CT provides *V. cholerae* an advantage over non-CT-expressing *Vibrio* during iron and branched-chain amino acid acquisition (32). Increased access to calcium ions also restricts *V. cholerae* production of the biofilm exopolysaccharide, *Vibrio* polysaccharide (33), and cholera biofilms are more virulent and outcompete planktonic counterparts in the suckling mouse colonization assay (34). We previously demonstrated that acute ST intoxication causes luminal iron and magnesium limitation in the small intestine (35), but it remains unclear if ETEC’s enterotoxins regulate access to luminal metabolites or ions that feedback onto the organism to regulate the course of its pathogenesis.

While ETEC have been identified in polymicrobial biofilms in household water storage tanks in Bangladesh (36, 37), a systematic and detailed analysis of ETEC biofilm formation remains unavailable. Previous studies have shown that the iron- and oxygen-sensing fumarate and nitrate reductase transcriptional regulator, FNR, suppresses ETEC biofilm formation (38), but the role of magnesium in ETEC biofilm formation remains unclear. Iron and magnesium limitation enhances CFA/I expression in ETEC H10407 (35, 39), but it remains unclear how ETEC adhere to the intestinal epithelium at basal conditions, when magnesium levels can reach as high as 10–20 mM (40) and are regulated by diet (41) and uptake into the sera.

Our findings demonstrate that ETEC can form robust biofilms at body temperature when cultured in a chemically defined media containing high levels of magnesium and lactate. Importantly, ETEC H10407 biofilms adhere to intestinal epithelial cells without expressing high levels of CFA/I. Our findings demonstrate that ETEC have evolved multiple mechanisms that allow it to adhere to the intestinal epithelium during different environmental conditions. Therefore, these data are significant in that ETEC biofilms represent an untapped and novel platform to identify antigens that may help develop vaccines to protect against ETEC-mediated secretory diarrheal disease.

## RESULTS

### Magnesium limitation induces CFA/I expression in diverse CFA/I-expressing ETEC

CFA-agar is typically used to grow ETEC strains for inoculation during controlled human challenge models (42). We previously demonstrated that ST intoxication causes luminal magnesium concentrations to drop from approximately 175 ppm (7.2 mM) at baseline to approximately 50 ppm (2 mM) 30 minutes following oral gavage, and reestablishment of the basal luminal magnesium concentration (7.2 mM) required approximately 90 minutes from a single oral gavage (35). We previously demonstrated that ST-mediated magnesium limitation may lead to induction of CFA/I fimbriae in ETEC H10407 (35), so we first explored whether magnesium limitation also induced CFA/I expression in other ETEC clinical strains isolated from patients enrolled in the GEMS (15).

The recipe for CFA-agar includes magnesium sulfate, so we prepared CFA-agar plates with and without magnesium sulfate and prepared lysates from ETEC isolates cultured from both conditions. Like ETEC H10407, our data demonstrate that magnesium limitation induces CFA/I expression in ETEC 300202, ETEC 300252, and ETEC 600880 (Fig. S1A and B). These data suggest that ST activity decreases luminal magnesium that leads to enhanced CFA/I expression.

### Magnesium-dependent ETEC H10407 biofilm formation

**Fig 1:**
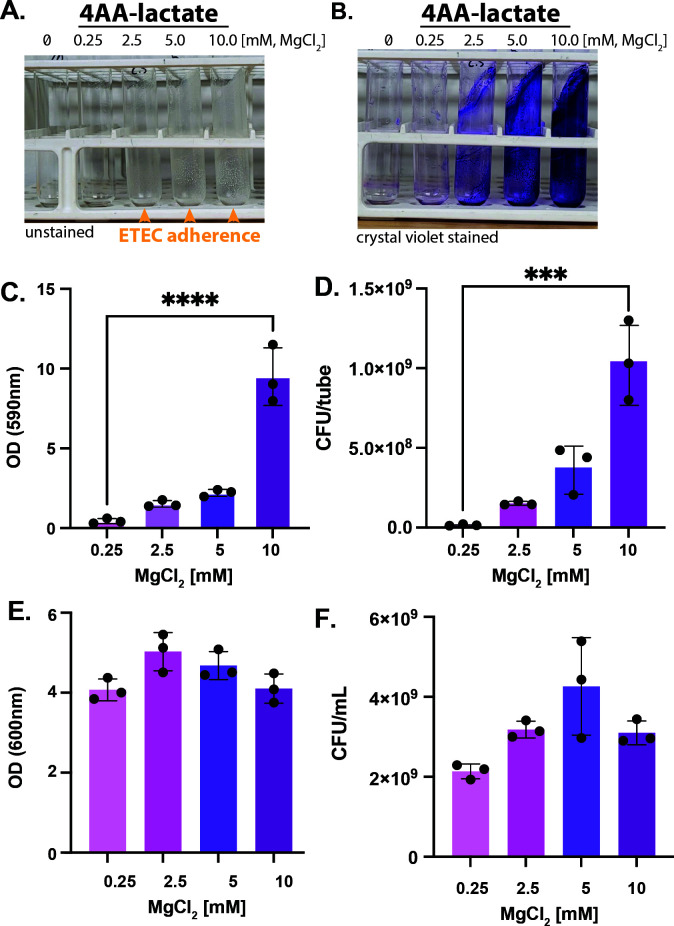
Magnesium induces ETEC H10407 biofilm formation. ETEC H10407 was grown in 4AA-lactate media containing 0, 0.25, 5, or 10 mM magnesium chloride at 37°C overnight at 250 rpm in a shaking incubator. The liquid phases of these cultures were then separated from the attached biofilm phase for quantifications. Representative photographs of ETEC H10407 grown in 13*100 mm borosilicate glass culture tubes demonstrate biofilm formation, as seen by the accumulation of biomass on the test tubes (**A**). ETEC H10407 from A were stained with crystal violet (0.1%) washed with PBS and photographed (**B**) and then eluted with ethanol to demonstrate magnesium-dependent increases in absorbance at 590 nm, indicative of increased ability to form biofilms with increased magnesium chloride concentration (**C**). Adherent biofilms were scraped from the culture tubes before being sonicated to disperse and count CFUs from each condition (**D**). The optical density at 600 nm of the planktonic phases of ETEC H10407 grown with different concentrations of magnesium chloride in 4AA-lactate media (**E**) and the CFUs of the planktonic phases of ETEC H10407 grown with different concentrations of magnesium chloride in 4AA-lactate media (**F**). Data were compiled from three independent experiments and analyzed via one-way ANOVA with Dunnett’s test for multiple comparisons; ***, *P* < 0.001; ****, *P* < 0.0001.

Magnesium concentrations can vary substantially in the human intestines (43), and ETEC are amenable to growth using a variety of conditions and growth media (7, 44). We previously adopted use of a chemically defined growth medium that enhances ST production (45). It is called 4AA-lactate because it is composed of a mixture of 4 amino acids (proline, aspartic acid, alanine, and serine) and uses lactate as a supplementary carbon source. It also contains basal salts and trace salts (containing magnesium), so we next set out to use 4AA-lactate media to understand if magnesium also regulates ST production. We grew ETEC H10407 in 4AA-lactate containing either 0.25 or 10 mM magnesium chloride to assess ST production and secretion. Interestingly, we found that ETEC H10407 produced and secreted less ST when cultured with 10 mM magnesium as compared to 0.25 mM magnesium (Fig. S1C). Although we have previously demonstrated that ETEC H10407 produces significantly more CFA/I when cultured on CFA-agar (as compared to 4AA-lactate), we now demonstrate that ETEC H10407 produces even less CFA/I when cultured with 10 mM magnesium as compared to 0.25 mM magnesium in 4AA-lactate (Fig. S1D). While this suggests that ETEC H10407 grown in high magnesium conditions would be less adherent, we found that the magnesium concentration of 4AA-lactate (0.25, 2.5, 5, and 10 mM magnesium chloride) directly correlates to ETEC H10407’s ability to form biofilms on the borosilicate glass culture tubes (Fig.1A). To quantify ETEC H10407 biofilm formation, we carefully removed the planktonic liquid phase and stained the tube-adherent biofilms with 0.1% crystal violet. As shown, increasing the magnesium concentration of 4AA-lactate media significantly enhances ETEC H10407’s ability to form tube-adherent biofilms (Fig. 1B). Biofilms were also scraped from the test tubes and plated onto Luria-Bertani (LB) agar to determine the colony-forming units (CFUs) at each magnesium concentration. As shown in Fig. 1C, we found that magnesium supplementation significantly increased the ability of ETEC H10407 to adhere to the test tubes and form biofilms. For comparison, we also show that the optical densities and CFUs of the aspirated planktonic cultures were not significantly different from each other, demonstrating that addition of magnesium did not restrict ETEC H10407’s ability to grow (Fig. 1E and F). To demonstrate that ETEC biofilm formation is broad, we selected ETEC isolates from a diverse collection with different CFs (46). Notably, magnesium induced biofilm formation in all of the selected isolates (Fig. S2).

### Visualization of the extracellular matrix of magnesium-dependent ETEC H10407 biofilms

**Fig 2:**
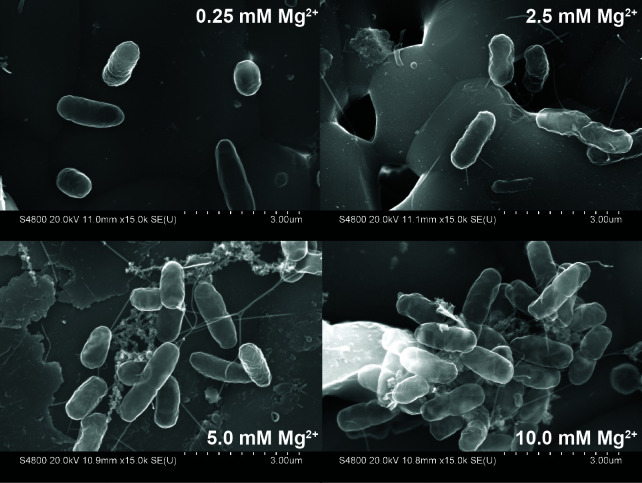
ETEC H10407 biofilms contain an extracellular polymeric matrix. Field emission scanning electron micrographs demonstrate ETEC H10407 biofilm formation on HA disks when grown in 4AA-lactate media containing either 0.25, 2.5, 5, or 10 mM magnesium chloride statically at 37°C for 24 hours. Images taken at 15,000× magnification. Scale bars represent 3 µm.

Next, we grew ETEC H10407 on hydroxyapatite (HA) disks in 4AA-lactate containing either 0.25, 2.5, 5, or 10 mM magnesium (Fig.2). As shown, ETEC H10407 grown at 0.25 mM magnesium featured many single and dividing ETEC with little extracellular structure. In contrast, ETEC H10407 grown at 2.5, 5, or 10 mM magnesium featured biofilm macrostructures and bacteria in close association with each other, encased in an extracellular matrix, especially at the highest magnesium concentration used. Extracellular polysaccharides, proteins, and DNA can give bacterial biofilms structure, so we began to assess the composition of ETEC H10407’s extracellular matrix when cultured in 4AA-lactate with 0.25 or 10 mM magnesium. We found that the addition of proteinase K at ≥8 µg/mL inhibited ETEC H10407 biofilm formation, indicating a significant protein component to the biofilm matrix (Fig. S3A). We also demonstrate that DNase I (10 µg/mL) prevented ETEC H10407 biofilm formation at similar concentrations to those used to prevent or disrupt *E. coli* biofilms (47, 48) (Fig. S3B).

### EDTA prevents ETEC H10407 biofilm formation

**Fig 3:**
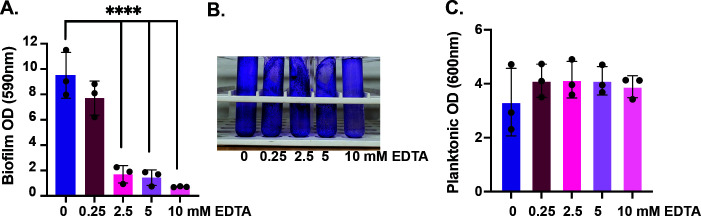
EDTA prevents magnesium-dependent biofilm formation in ETEC H10407. The divalent metal ion chelator EDTA was titrated at 0, 0.25, 2.5, 5, or 10 mM into overnight cultures of ETEC H10407 growing in 4AA-lactate with 10 mM magnesium chloride. The biofilm phases of these cultures were quantified by 0.1% crystal violet staining (**A**). Representative image of crystal violet-stained culture tubes following EDTA titration (**B**). Planktonic phases were quantified by measuring optical densities (**C**). Data were compiled from three independent experiments and analyzed via one-way ANOVA with Dunnett’s test for multiple comparisons; ****, *P* < 0.0001.

Since we found that magnesium promotes ETEC H10407 biofilm formation in 4AA-lactate, we designed a magnesium chelation experiment to show that the additional chloride added in the form of magnesium chloride was not affecting ETEC biofilm formation. This is important because we and others previously demonstrated that sodium chloride suppresses CFA/I expression in ETEC H10407 (35, 49). Therefore, we titrated the divalent metal ion chelator, EDTA (0, 0.25, 2.5, 5, and 10 mM), into 4AA-lactate containing 10 mM magnesium chloride and quantified ETEC H10407 biofilm formation following overnight growth. As shown, when EDTA is included at a concentration of 2.5 mM or above, ETEC biofilm formation is significantly decreased, compared to culture grown in its absence (Fig. 3A and B). Importantly, EDTA does not restrict planktonic growth of ETEC H10407 at any of the concentrations tested (Fig. 3C). This indicates that magnesium regulates biofilm formation in ETEC H10407. Since EDTA is not physiologically relevant, we also tested the ability of the divalent metal-chelating metabolite citrate to inhibit ETEC H10407 biofilm formation. As shown in Fig. S4A, when citrate is included at a concentration of 4.5 mM or above, ETEC biofilm formation is significantly decreased, compared to controls. Importantly, citrate at these concentrations does not impact ETEC H10407 planktonic growth (Fig. S4B). While magnesium sensing can be regulated by the PhoPQ two-component system in enteric pathogens (50), the PhoP regulon has not been characterized in ETEC. However, the PhoPQ regulon has been established for commensal *E. coli* (51) and uropathogenic strains (52), which helped us define a putative Pho box (5′-TTTTGATTTATTATTGA-3′) 20 nucleotides upstream of the ATG start codon for *cfaA* in the CFA/I operon.

### Role of lactate on ETEC H10407 biofilm formation

**Fig 4:**
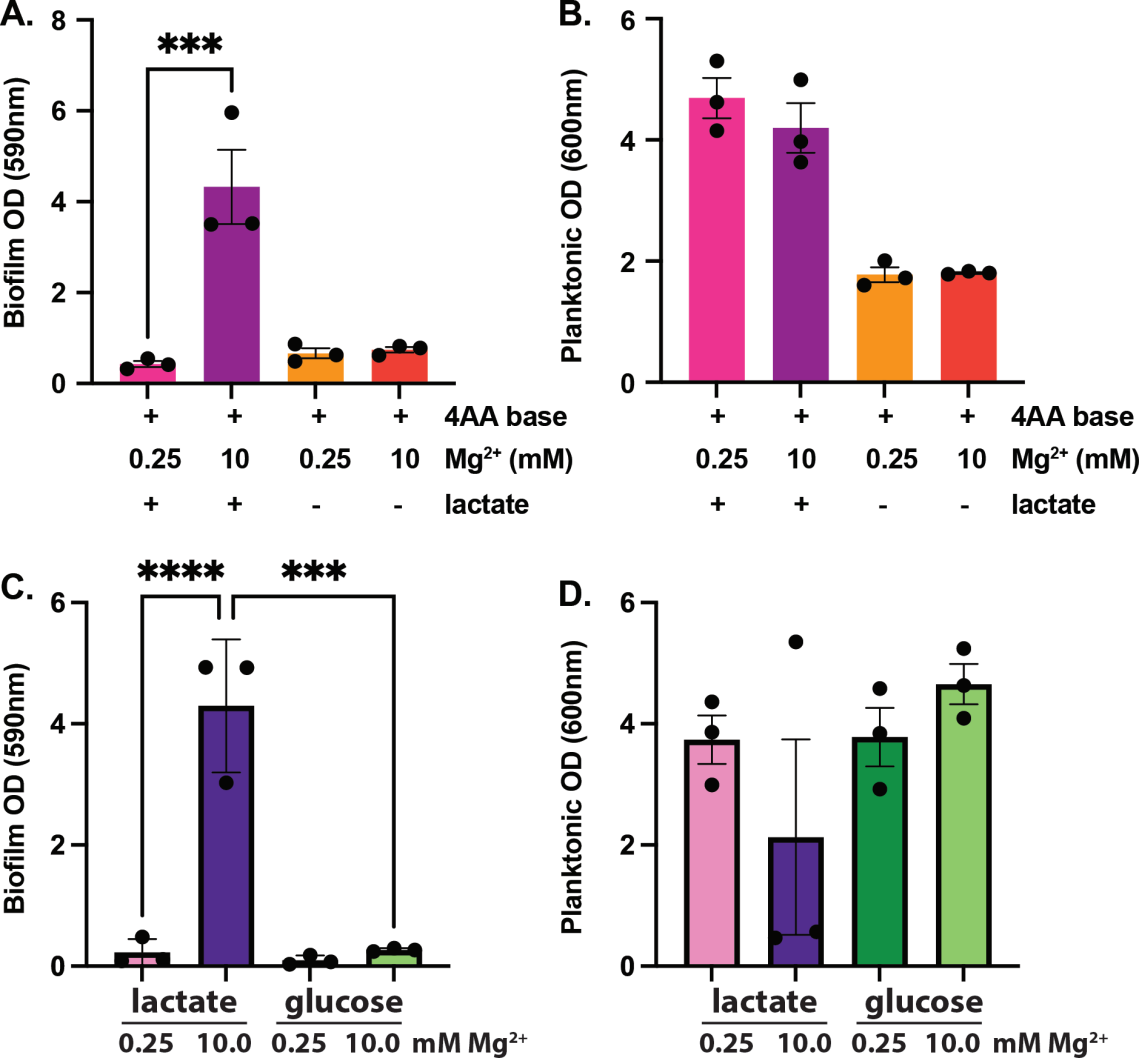
ETEC H10407 biofilm development requires lactate. ETEC H10407 was cultured in 4AA with or without lactate, and the crystal violet-stained attached biofilm phase (**A**) and liquid phase optical density (**B**) were measured. Similarly, ETEC H10407 was cultured in 4AA with either lactate or glucose. Biofilm formation in these conditions was quantified by crystal violet staining (**C**), and the liquid phase was assessed by measuring optical density (**D**). Data are compiled from three independent experiments and analyzed via one-way ANOVA with Tukey’s test for multiple comparisons; ***, *P* < 0.001; ****, *P* < 0.0001.

As mentioned, ST is induced by gluconeogenic carbon sources including lactate (45), but ETEC’s preferred carbon source *in vivo* remains unclear. Recent studies have demonstrated that lactate catabolism promotes virulence of other enteric pathogens including *Salmonella enterica* serovar Typhimurium (53) and *Campylobacter jejuni* (54). Therefore, we reasoned that ETEC’s ability to form biofilms in 4AA-lactate could be a unique phenomenon based on access to lactate. We cultured ETEC H10407 in 4AA with or without lactate and demonstrate that lactate is required for biofilm formation (Fig. 4A). It is worth noting that ETEC H10407 can grow planktonically in 4AA media without lactate supplementation (Fig. 4B), albeit to lower ODs. Since 4AA media is amenable to different carbon sources, we next assessed the ability of ETEC H10407 to form magnesium-dependent biofilms in 4AA containing either lactate or glucose at molar equivalent concentrations of carbon. In contrast to the robust ETEC H10407 biofilms formed in 10 mM magnesium-containing 4AA-lactate, we show that ETEC H10407 does not form biofilms when grown in 4AA-glucose, regardless of the magnesium concentration (Fig. 4C). However, ETEC H10407 was able to grow planktonically in 4AA-lactate and 4AA-glucose (Fig. 4D).

### ETEC biofilms are more resistant to acid stress than planktonic ETEC

**Fig 5:**
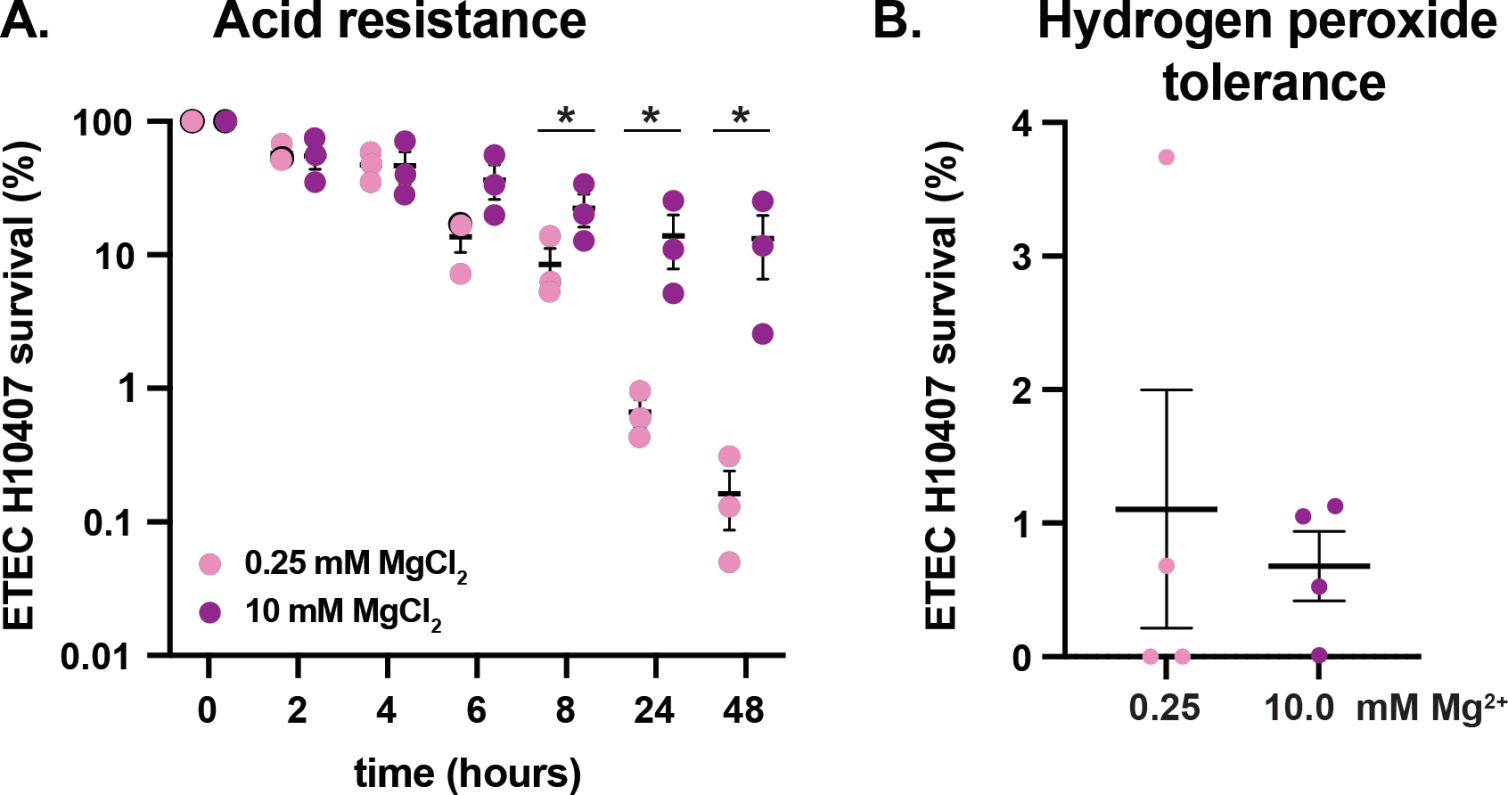
Acid and hydrogen peroxide tolerance of ETEC H10407 biofilms. ETEC H10407 was grown in 4AA-lactate with 10 mM magnesium chloride to induce biofilm formation or in 4AA-lactate with 0.25 mM magnesium chloride for planktonic conditions. Biofilm and planktonic cultures were resuspended in pH 2.8 glycine buffer and incubated for up to 48 hours. The cultures were then serially diluted and plated to enumerate initial CFUs and surviving CFUs at 2, 4, 6, 8, 24, and 48 hours of exposure (**A**). Biofilm and planktonic cultures were incubated in 50 mM hydrogen peroxide for 15 minutes and then also diluted and plated for enumeration (**B**). Data were compiled from three (acid tolerance) or four (peroxide tolerance) independent experiments and analyzed via Student’s *t*-test at each time point; *, *P* < 0.05.

Biofilms typically provide organisms resistance to antimicrobial, acid, or oxidative stress. As fecal-oral pathogens, ETEC must adapt to a variety of environmental niches and must have developed mechanisms that allow it to survive transit through gastric acidity so that it can reach the small intestines. Interestingly, we show that ETEC H10407 cultured in either 0.25 or 10 mM magnesium have similar starting and ending pHs, indicating that pH sensing is unlikely to induce magnesium-dependent biofilm formation (Fig. S4C). Thus, we next investigated whether biofilms could provide ETEC with enhanced resistance to low pH using a glycine buffer (55). As shown, ETEC H10407 biofilms (grown in 4AA-lactate with 10 mM magnesium) are significantly more resistant than planktonic ETEC H10407 (grown in 4AA-lactate with 0.25 mM magnesium) to acid killing after 8 hours (Fig. 5A). Since ETEC are closely juxtaposed to intestinal epithelial cells, they could also be subjected to host-mediated oxidative stress. Therefore, we compared the sensitivity of ETEC H10407 biofilms and planktonic cultures to hydrogen peroxide. As compared to planktonic ETEC H10407, biofilms did not provide a benefit to better tolerate oxidative damage induced by hydrogen peroxide (Fig. 5B).

### ETEC biofilms attach to intestinal epithelial cells

**Fig 6:**
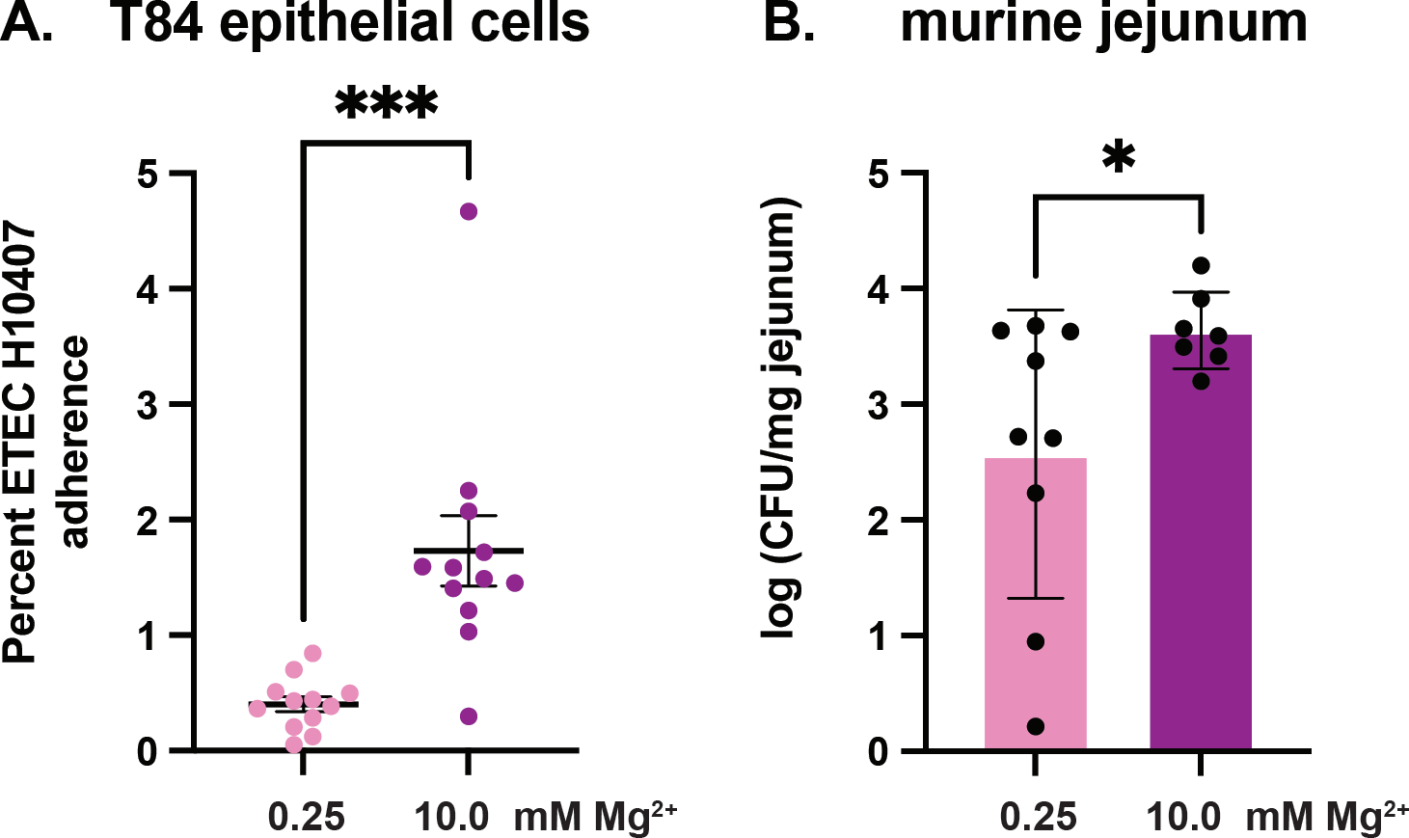
Adherence of ETEC H10407 biofilms to epithelial cells. ETEC H10407 was grown in 4AA-lactate with 10 mM magnesium chloride to induce biofilm formation or 4AA-lactate with 0.25 mM magnesium chloride for planktonic conditions. ETEC H10407 biofilms and planktonic ETEC H10407 were resuspended and applied to T84 cells at 10^6^ CFUs per well. Wells were then washed to remove unattached bacteria, and remaining cells and bacteria were resuspended and plated for CFUs (**A**). CD1 mice were given streptomycin to clear their normal flora and then orally infected with 10^8^ CFU ETEC. Twenty-four hours post-infection, the jejunal tissue of the mice was harvested and processed to plate for CFUs (**B**). T84 data are compiled from three independent experiments. Mouse data are compiled from two independent experiments in which a total of nine mice were dosed with low magnesium cultured ETEC H10407 and seven were dosed with 4AA-lactate 10 mM magnesium chloride biofilm ETEC H10407. Data were analyzed by *t*-test; *, *P* < 0.05; ***, *P* < 0.001.

Although it is well-known that CF fimbriae provide ETEC with the ability to attach to the intestinal epithelium, more than half of the sequenced ETEC isolates fail to express major CF antigens (15). Non-canonical ETEC antigens may also provide ETEC with the ability to adhere to enterocytes (19, 56), but it remains unclear if biofilms provide ETEC H10407 with a colonization advantage. To assess the importance of biofilm antigens in mediating adherence, we compared the ability of ETEC H10407 biofilms (grown in 4AA-lactate containing 10 mM magnesium) and planktonic ETEC H10407 (grown in 4AA-lactate containing 0.25 mM magnesium) to adhere to T84 intestinal epithelial cell monolayers. Interestingly, we found that ETEC H10407 biofilms were significantly better than planktonic ETEC H10407 at adhering to T84 cells (Fig. 6A). Next, we sought to evaluate if ETEC H10407 biofilms were better than planktonic ETEC at establishing an infection in mice that were orally infected in the streptomycin mouse model. As shown, we found the jejunum of mice infected with ETEC H10407 biofilms had significantly more ETEC than mice who were infected with planktonic H10407 (Fig. 6B). In support of the overarching premise that enterotoxins may alter luminal metabolites to promote different mechanisms of ETEC adherence, we finally demonstrate that ST intoxication enhances the ability of ETEC to colonize T84 cells while also suppressing the ability of *Citrobacter rodentium* to colonize T84 cells (Fig. S5). This suggests that enterotoxins may be deployed to regulate the balance of ETEC and competitor species colonizing a respective niche.

### ETEC H10407 biofilms are more lethal in neonatal mice

**Fig 7:**
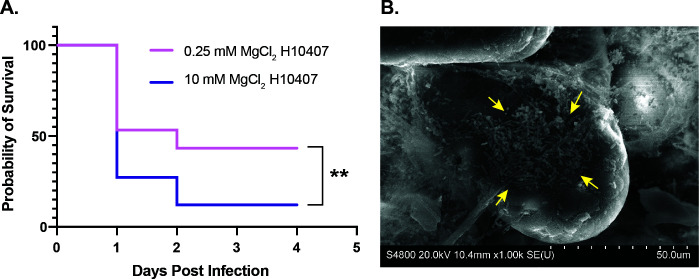
Lethality of ETEC H10407 biofilms in a murine neonatal challenge model. Neonatal mice were infected intragastrically with 10^7^ CFUs of ETEC H10407 grown in 4AA-lactate containing 0.25 or 10 mM magnesium chloride and monitored for up to seven days (**A**). Small intestinal samples from infected pups were examined by scanning electron microscopy, and a dense patch of ETEC was identified on a murine villue following biofilm inoculation. Pictured at 1000× magnification, scale bar represents 50 µm (**B**). Data were compiled from three independent experiments featuring pups from six different dams. In total, 30 1- to 3-day-old pups were administered planktonic ETEC H10407 grown in 4AA-lactate with 0.25 mM magnesium chloride, and 33 pups were administered ETEC H10407 biofilms grown in 4AA-lactate with 10 mM magnesium chloride. Survival was analyzed by Mantel-Cox test; **, *P* < 0.01.

While peroral infection of adult mice is useful to establish ETEC shedding rates in feces, when ETEC are quantified from small intestines, it is difficult to distinguish if ETEC are attached to the epithelium or if they are residing planktonically in the lumen. Therefore, to truly evaluate the role of biofilms in ETEC pathogenesis, we used the neonatal challenge model using 1- to 3-day-old CD1 pups (57). We found that ETEC H10407 biofilms result in more severe disease as compared to planktonic ETEC H10407 (Fig. 7A). Finally, we harvested the ileum from 1- to 3-day-old pups intragastrically inoculated with ETEC biofilms or planktonic cultures and mounted samples for SEM. While we were able to find adherent ETEC following infection with ETEC H10407 biofilms (Fig. 7B, detail Fig. S6), we were unable to locate adherent ETEC following infection with planktonic ETEC H10407.

## DISCUSSION

ETEC can be shed into the stools of infected individuals and oftentimes make their way into surface waters (e.g., Choqueyapu River in Bolivia) (58) that is used for drinking, washing, bathing, and cooking in LMICs (59). After being shed into the environment, ETEC must persist before being consumed by a susceptible host, upon which ETEC must be armed with defenses that enable its passage through gastric acidity before it reaches its permissive site of infection. It is reasonable to suspect that ETEC have evolved multiple mechanisms that allow it to persist both inside and outside of the gastrointestinal tract. Many wild ETEC isolates can produce both the ST and LT enterotoxins, but either alone is sufficient to cause secretory diarrheal disease. While the evolutionary benefit conferred by producing both enterotoxins remains unclear, our data indicate that the enterotoxins may provide access to host metabolites and ions that allow ETEC to colonize the small intestinal niche.

We have demonstrated that ETEC H10407 and other ETEC isolates form robust biofilms at body temperature. ETEC have been identified in biofilms attached to drinking water reservoirs (36), but systematic analysis of ETEC biofilm formation is lacking. Here, we demonstrate that ETEC biofilm formation requires 4AA base media containing lactate and high levels of magnesium. Magnesium limitation can induce CFA/I fimbriae expression in ETEC H10407 (35), but also elevated magnesium can induce CS3 (60) and CS21 (61) expression in other ETEC isolates (e.g., ETEC E9034A). These contrasting data demonstrate that the transcriptional responses among ETEC strains are different, but it may not be surprising that ETEC regulate different CFs depending on host signals. A CFA/I-deficient ETEC H10407 *cfaE* knockout showed impaired attachment to intestinal epithelia *in vitro*, demonstrating that CFA/I is important for ETEC attachment during some conditions (62). However, it is worth noting that expression of CFA/I’s major structural subunit gene, *cfaB*, is lower in ETEC-infected stool samples from human volunteers as compared to laboratory CFA media-grown conditions (38). Importantly, we found that magnesium-dependent ETEC H10407 biofilms cultured in 4AA-lactate lack the high levels of CFA/I fimbriae found when cultured on CFA-agar but still adhere to intestinal epithelial cells robustly. These data suggest that access to magnesium and lactate is required to promote ETEC H10407 biofilm formation under our experimental conditions.

*E. coli* typically sense magnesium via the PhoQ/PhoP two-component system (63). PhoQ is a membrane-bound sensor of extracytoplasmic magnesium that modifies the phosphorylation state of the DNA-binding protein PhoP (50). When magnesium levels are low (micromolar range), PhoP activates expression of target genes that participate in magnesium import (e.g., *mgtA*), lipopolysaccharide (LPS) modification (e.g., *ugd*) (52), and acid tolerance (e.g., *gadA* and *gadY*) (50). While the genes responsible for increased acid tolerance of ETEC H10407 biofilms remain unclear, deamination of L-serine may also play a role since serine is provided in the components of 4AA (64). However, while it is typically thought that the PhoP regulon is repressed when magnesium levels are high (>2 mM), studies carried out in uropathogenic *E. coli* (UPEC) show that PhoP also senses membrane potential (52). A high magnesium concentration (like the one found in the gut) could electrostatically quench LPS’s net negative charge, activate PhoP, and allow ETEC to coalesce and promote biofilm formation. Indeed, PhoP regulates the virulence of other enteric pathogens similarly susceptible to the high levels of magnesium found in the gut. PhoP is required for *Salmonella* survival in macrophages (65, 66), epithelial invasion of *Shigella flexneri* (67), and expression of genes at the locus of enterocyte effacement (lee) pathogenicity island in enterohemorrhagic *E. coli* (EHEC) (41*).*

L-lactate can be sensed by the LldR transcriptional regulator, and while the transcriptional response to lactate has been defined for commensal *E. coli* (68), it is unclear how lactate regulates ETEC biofilm formation and virulence. Lactate concentrations in the lumen of the colon only reach ~5 mM because of the presence of lactate-utilizing bacteria (69), but lactate concentrations in the lumen of the small intestines can reach upward of 50–100 mM (70). Lactate can be produced by enterocytes when oxygen tension is reduced (53) or as a metabolic end product by the members of the microbiome (e.g., lactobacillus, streptococcus, and bifidobacteria) (71). An elevated lactate concentration promotes the fitness of *Salmonella* spp. (72), (non-ETEC) *E. coli* (73), and *C. jejuni* (54) in the inflamed colon. However, it remains unclear if lactate promotes ETEC biofilm formation in the small intestines. Paneth cells also export lactate to promote proliferation of intestinal stem cells (74), so lactate-sensing may allow ETEC to settle in the small intestinal niche. We are currently evaluating the overlapping transcriptional responses induced by magnesium and lactate that lead to production of ETEC biofilms.

We previously demonstrated that ST intoxication causes a decrease in luminal magnesium levels (35), so it remains plausible that ETEC may encode enterotoxins to gain access to host metabolites that allow outcompete competitor species. How the host responds to enterotoxins remains to be fully evaluated, but these data demonstrate that enterotoxin-mediated changes in luminal magnesium could be sensed by ETEC strains to regulate how it adheres to the intestinal epithelium.

Biofilms may mask ETEC by decreasing the expression of CFs and canonical ETEC virulence factors, which may contribute to key differences in the ability of a particular strain to result in symptomatic or asymptomatic infection. Therefore, systematic identification of conserved ETEC biofilm antigens may be required to develop a broadly protective ETEC vaccine. Although ETEC biofilms remain relatively uncharacterized, biofilms from other *E. coli* contain protein and exopolysaccharide components including curli, cellulose, colonic acid, and polysialic acid (75). Curli expression is observed broadly across ETEC isolates of diverse geographic origin and virulence factor profile (76), and the biofilm regulator diguanylate cyclase DgcC can activate cellulose synthase complex (77). While the ability to induce anti-CF immunity remains an important aspect of ETEC vaccines, the development of anti-biofilm immunity may be just as important. ETEC’s ability to switch between multiple adherence mechanisms may have allowed them to subvert mucosal immunity in infected individuals.

While we performed this study using monocultures of ETEC, future assessment of multi-species biofilms containing lactic acid-producing bacteria may help us understand the role of the microbiome on providing cues that allow ETEC strains to transition between CF- and biofilm-mediated adherence. Strain-specific responses to environmental signals like magnesium and lactate still need to be parsed to determine the breadth of mucosal signals that promote ETEC biofilms.

## MATERIALS AND METHODS

### Bacterial strains and culture conditions

ETEC strains, H10407, H10407p, 300202, 300252, 600880, 214-4, 503025, and 510016 were streaked for isolation onto LB agar from a −80°C glycerol stock and grown overnight at 37°C. Isolated single colonies were inoculated into the chemically defined 4AA base media containing lactate (4AA-lactate) or glucose (4AA-glucose) (78). Cultures were adapted to each media in a 37°C incubator shaking at 250 RPM overnight. The next morning, the bacteria were sub-cultured 1:100 in fresh 4AA-lactate or 4AA-glucose containing different concentrations of magnesium chloride, EDTA, citrate, DNase I, and/or proteinase K. *C. rodentium* (ATCC, 51459) was cultured in LB broth when used.

### Planktonic and biofilm measurement

Cultures were grown in a 37°C incubator shaking at 250 RPM overnight; then, the planktonic fraction, containing the spent medium and non-adhered bacteria, was collected and diluted; and the absorbance at 600 nm was measured using a BioTek spectrophotometer. Subsequently, culture tubes were then photographed and gently washed to remove loosely bound bacteria cells before the addition of 0.1% crystal violet for 30 minutes followed by a washing step. Culture tubes were dried by inversion at room temperature overnight, and crystal violet-stained biofilms were solubilized using 90% ethanol for 30 minutes. Biofilms were quantified via measuring the absorbance at 590 nm on the spectrophotometer.

### Scanning electron microscopy

Scanning electron microscopy was carried out in the Tulane Instrumentation for Nanoscience and Innovation laboratory. HA disks were placed in wells of 24-well plates containing 4AA-lactate with 0.25, 2.5, 5, or 10 mM magnesium chloride. Overnight bacterial cultures were sub-cultured into the wells and allowed to grow at 37°C for 24 hours. HA disks were removed from the growth media and washed three times with phosphate buffered saline (PBS), and then, the HA disks were placed into wells containing 2.5% glutaraldehyde for fixation for 24 hours at 4°C. The HA disks were then washed three times with H_2_O (10 minutes each) and then dehydrated stepwise with increasing concentrations of ethanol such that disks were placed for 5 minutes in 25% ethanol, then 5 minutes in 50% ethanol, then 5 minutes in 75% ethanol, then 5 minutes in 90% ethanol twice, and then 5 minutes in absolute ethanol thrice. HA disks were maintained in absolute ethanol for storage and transport to the microscopy facility. HA disks were dried at the critical point of CO_2_ using a Tousimis Autosamdri-810 before applying a thin layer of carbon using a Cressington 208C carbon coater. Scanning electron micrographs were acquired using a Hitachi S-4800 microscope. At least five fields of view were recorded for each condition shown.

### T84 cell adherence

T84 human colonic epithelial cells (CCL-248) were purchased from ATCC and cultured on 24-well tissue culture-treated plates in Dulbecco’s Modified Eagle’s Medium (DMEM) supplemented with 5% fetal bovine serum. Cells were allowed to grow to ≥80% confluency before use in bacteria adherence experiments. T84 cells were washed, and media was replaced with serum-free, antibiotic-free DMEM. Cells were allowed to rest for 1 hour, and then, ETEC H10407 (10^6^ CFU) grown planktonically or in biofilms was applied to the monolayers. T84 cells were also treated with and without ST, and 10^4^ CFUs ETEC H10407 or *C. rodentium* was applied at 37°C for 1 hour to attach. For each experiment, the media was removed, and the wells were washed three times with sterile PBS to remove non-adherent bacteria. Wells were then incubated for 10 minutes with 0.1% Triton-X to facilitate removal of T84s from the surface, and dilutions of the wells were plated in triplicate to quantify attachment.

### Acid and hydrogen peroxide tolerance assays

Planktonic ETEC H10407 and biofilms were harvested and adjusted to a target starting point of 5 × 10^8^ CFU/mL by OD_600_ and pelleted by centrifugation at 18,000 relative centrifugal force (RCF) for 3 minutes. For assessing acid tolerance, the supernatant was removed, and the pellet was then resuspended in 0.1 M glycine buffer (pH = 7.0) and pelleted again. The supernatant was removed, and the pellet was resuspended in 0.1 M glycine buffer (pH = 2.8). Initial assessments of starting CFUs were taken by serially diluting this suspension and plating for CFUs. Bacteria were allowed to continue incubating in the acidic buffer statically at room temperature, and assessments of surviving CFUs were made periodically. For assessing hydrogen peroxide tolerance, the supernatant was removed, and the pellet was then resuspended in sterile PBS with 50 mM H_2_O_2_. Initial assessments of starting CFUs were taken by serially diluting this suspension and plating for CFUs. Bacteria were allowed to incubate for 15 minutes shaking at 250 RPM and 37°C and then serially diluted to assess surviving CFUs.

### Streptomycin ETEC murine colonization model

For the assessment of ETEC gut colonization, we used a streptomycin-water mouse model previously described (28). Female CD1 mice aged 7–9 weeks were obtained from Charles River Laboratories. Mice were allowed to adapt to their new housing for a minimum of 72 hours before use. Normal flora was depleted by providing the mice 5 g/L streptomycin in their drinking water 48 hours prior to infection, and then, streptomycin drinking water was replaced with normal water 24 hours before infection. Food was withdrawn 18 hours prior to infection. Mice were infected orally with 10^8^ CFUs ETEC H10407 biofilms or planktonic ETEC H10407. Mice were euthanized 24 hours post-infection, and small intestinal tissue was collected and serially diluted to plate for CFUs.

### Neonatal mouse lethal ETEC challenge model

The neonatal mouse ETEC challenge model has been used to compare the lethality of bovine, porcine, and human ETEC isolates (57). The CD1 mouse strain was selected for use here based on susceptibility to ETEC H10407 and low incidence of cannibalism. One- to three-day-old CD1 pups were intragastrically inoculated with 20 µL containing 10^7^ CFUs of ETEC H10407 grown in 4AA-lactate containing either 0.25 or 10 mM magnesium chloride using ultrathin needles. CFUs in each inoculum were confirmed by plating serial dilution onto LB agar plates. Bacterial inoculation was facilitated by presence of the milk in the stomachs of neonates. Pups were monitored daily following infection to check survival, and neonates that died within 0.5–2 days after ETEC challenge were considered in the determination of mortality rates. No more pups showed deteriorating symptoms after 2 days post-challenge.

### ST ELISAs

Competitive ST enzyme-linked immunosorbent assays (ELISAs) with monoclonal anti-ST antibodies were carried out. ELISA coating antigens were prepared via glutaraldehyde conjugation of STh to ovalbumin. Microtiter plates were coated overnight with 0.1 µg per well of ST-Ova conjugate in ELISA buffer (15 mM Na_2_CO_3_, 34.9 mM NaHCO_3_, 3 mM NaN_3_, pH 9.6). Cell-free supernatants from ETEC cultures were pre-incubated with a monoclonal ST antibody (Biosynth M120530) at a 1:16,000 dilution before being added at 1:2 dilutions down respective columns of the ST-Ova coated plate. They were then incubated for at least 2 hours at room temperature or 4°C overnight before washing the plates and incubating with rabbit anti-mouse IgG-AKP conjugate (Sigma A1902, St. Louis, MO, USA), which was used as a secondary antibody at a 1:400 dilution for 1 hour at room temperature. Plates were developed for 30 minutes to 1 hour with 0.1 mL of freshly prepared developing buffer (1 mg/mL para-nitrophenol phosphate (Sigma, N2765) dissolved in 9.7% diethanolamine, 0.5 mM MgCl2, pH 9.8). Absorbance at 405 nm was read using a Bio-Tek plate reader.

### Western blots

Purified CFA/I (NR-49109) was obtained from BEI Resources and used as positive controls for Western blots. Clarified ETEC lysates were loaded at equal mass onto a 10% polyacrylamide gel and run at 160 V for 35 min and then transferred to a nitrocellulose membrane using the iBlot 2. The membrane was blocked with 5% skim milk in 0.05% PBST and then probed with rabbit antisera against CFA/I (1:3,000) (courtesy of Dr. Eileen Barry, University of Maryland). The secondary antibody (1:2,500) was a goat-produced anti-rabbit IgG-HRP conjugate (Southern Biotech 1040-05, Birmingham, AL, USA) developed using a peroxidase substrate solution (Thermo Scientific 32209). The Western blots were imaged using an Amersham Imager 600, and post-image densitometry analysis was carried out using ImageJ (version 1.53 m).

### Statistical analysis

Statistical analyses were carried out using Prism 10 (GraphPad). *P* values < 0.05 were considered significant. For comparing titrations of magnesium chloride or EDTA, one-way ANOVA with Dunnett’s test for multiple comparisons to compare each condition to a baseline. For comparison of 4AA-lactate with 10 mM magnesium’s induction of biofilms across different strains, two-way analysis of variance (ANOVA) with Šídák’s multiple comparison post-test was used to determine statistical significance between different conditions of each isolate. The statistical significance of the Kaplan-Meier survival curve was calculated with a Mantel-Cox test.

## SUPPLEMENTAL MATERIAL

Supplemental figures iai.00243-25-s0001.pdf Fig. S1 to S6.

ASM does not own the copyrights to Supplemental Material that may be linked to, or accessed through, an article. The authors have granted ASM a non-exclusive, world-wide license to publish the Supplemental Material files. Please contact the corresponding author directly for reuse.
