# Supplementary material for: ETEC biofilms are regulated by magnesium and lactate bioavailability
Source: Infect Immun. 2025 Aug 11;93(9):e00243-25. doi: 10.1128/iai.00243-25 (PMC12418743; doi:10.1128/iai.00243-25)
Supplement: Supplemental figures — Fig. S1 to S6. [file iai.00243-25-s0001.pdf]

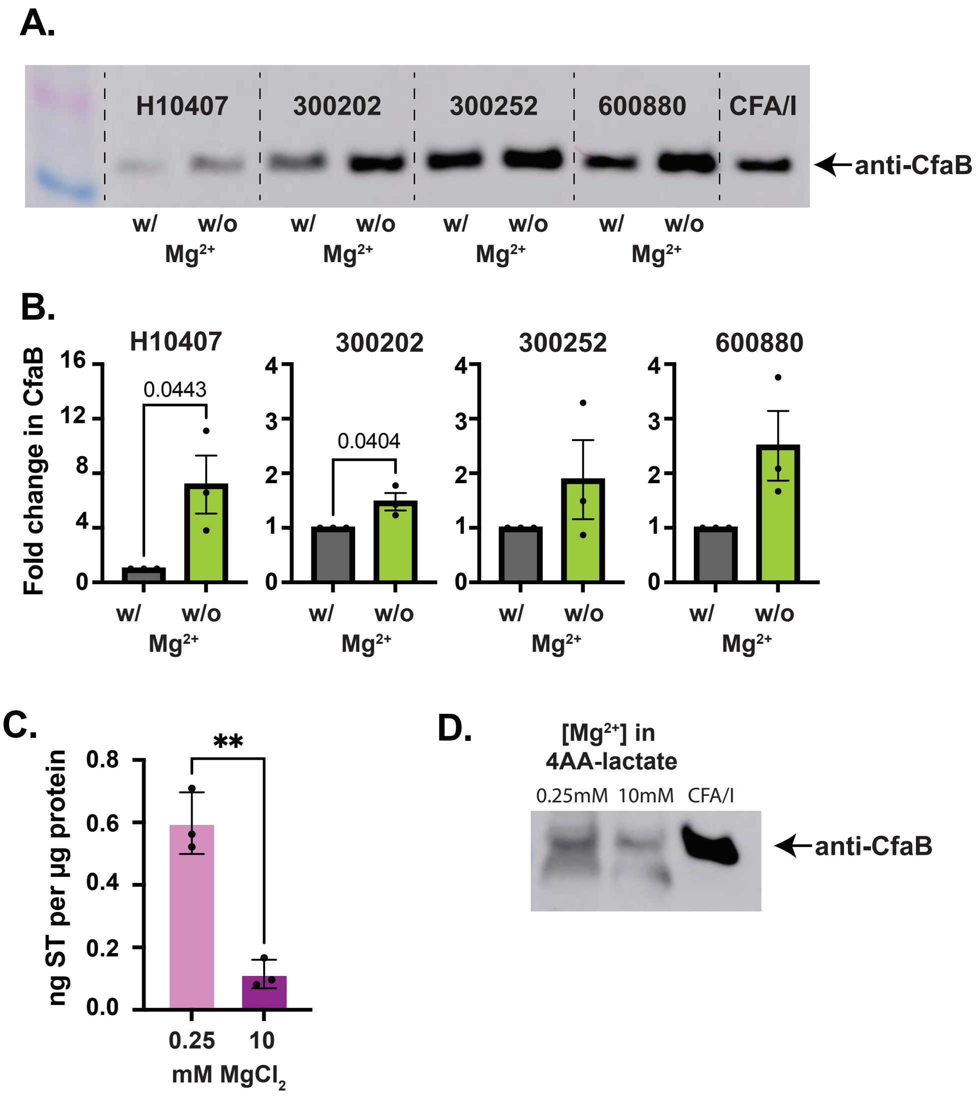

Fig. S1

Figure S1: Magnesium restricts production of CFA/I and ST. ETEC strains H10407, 300202, 300252, and 600880 were grown and harvested from CFA-agar plates supplemented with and without magnesium. Five micrograms of each lysate was loaded onto a reducing SDS-PAGE gel and transferred to a PVDF membrane for Western blotting using an anti-CFA/I polyclonal antibody (provided by Dr. Eileen Barry). Purified CFA/I (200 ng) was acquired from BEI Repositories (cat # NR-49110) and used as a positive control. (A) ImageJ densitometry analysis of at least 3 independent western blots shown in A (B). ST quantification in supernatants from ETEC H10407 grown in 4AA-lactate with 0.25- or 10 mM magnesium chloride measured via an ST-competitive ELISA using a monoclonal anti-ST antibody (C). Five micrograms of total ETEC H10407 lysates grown in 4AA-lactate with 0.25 mM or 10 mM magnesium chloride were loaded onto a reducing SDS-PAGE gel and transferred to a PVDF membrane for Western blotting using an anti-CFA/I polyclonal antibody (D). Data in B-C were compiled from three independent experiments and analyzed by t-test; **, $p<0.01$.

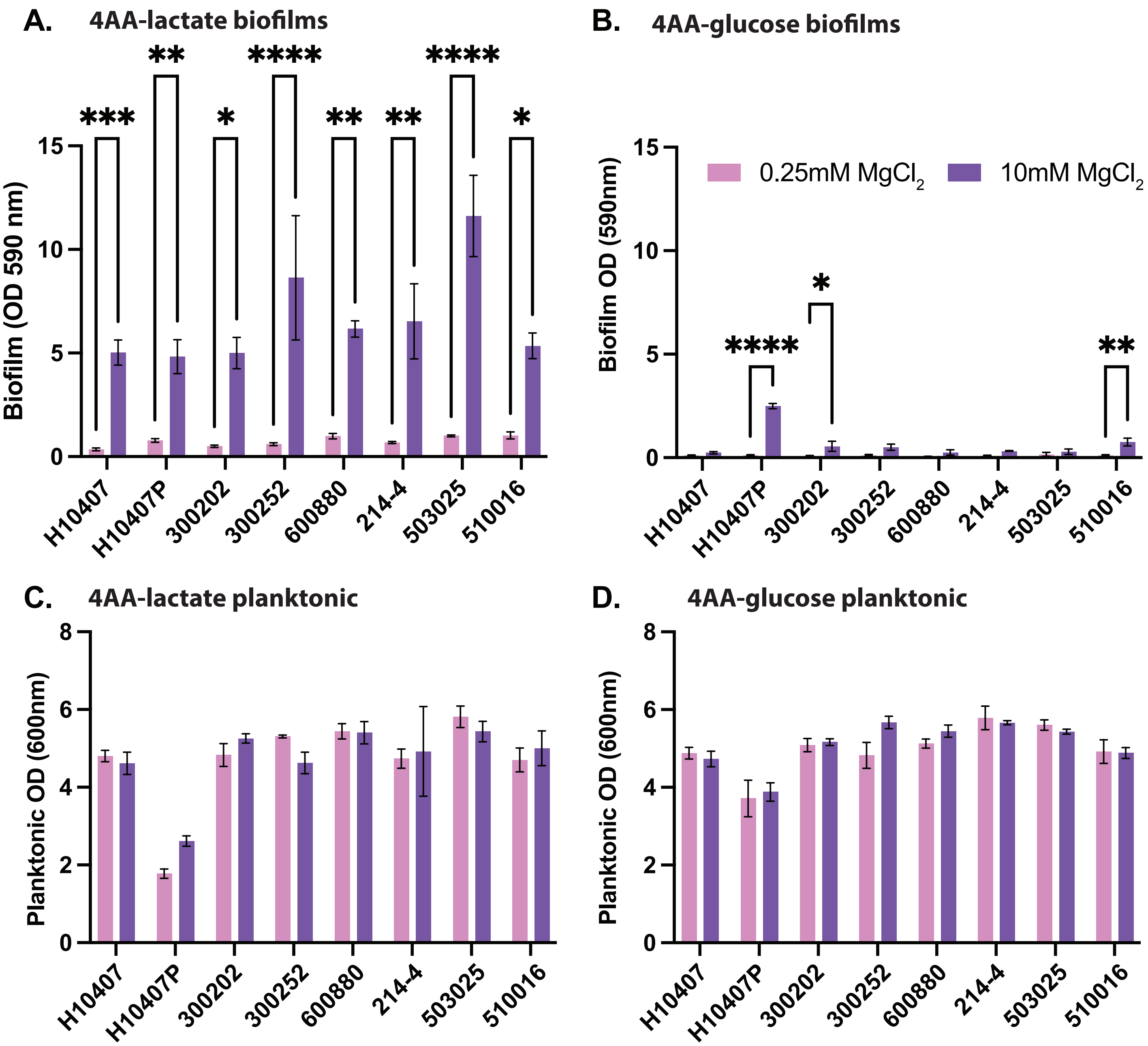

Fig. S2

Figure S2: Magnesium and lactate induce biofilm formation in diverse ETEC strains. ETEC strains H10407, H10407P, 300202, 300252, 600880, 214-4, 503025, 510016 were grown in 13*100 mm glass culture tubes in 4AA-lactate (A, C) with 0.25 mM or 10 mM magnesium chloride or 4AA-glucose (B, D) with 0.25 mM or 10 mM magnesium chloride. The planktonic phase was physically separated from the biofilm phase for quantifications. Biofilm phases were gently washed before ETEC biofilms were quantified via crystal violet staining (A, B). Planktonic phases were quantified by measuring optical densities (B,D). Data are compiled from three to six replicates per isolate and analyzed by two-way ANOVA with Šídák's test for multiple comparisons; *, $p < 0.05$; **, $p < 0.01$, ****, $p < 0.0001$.

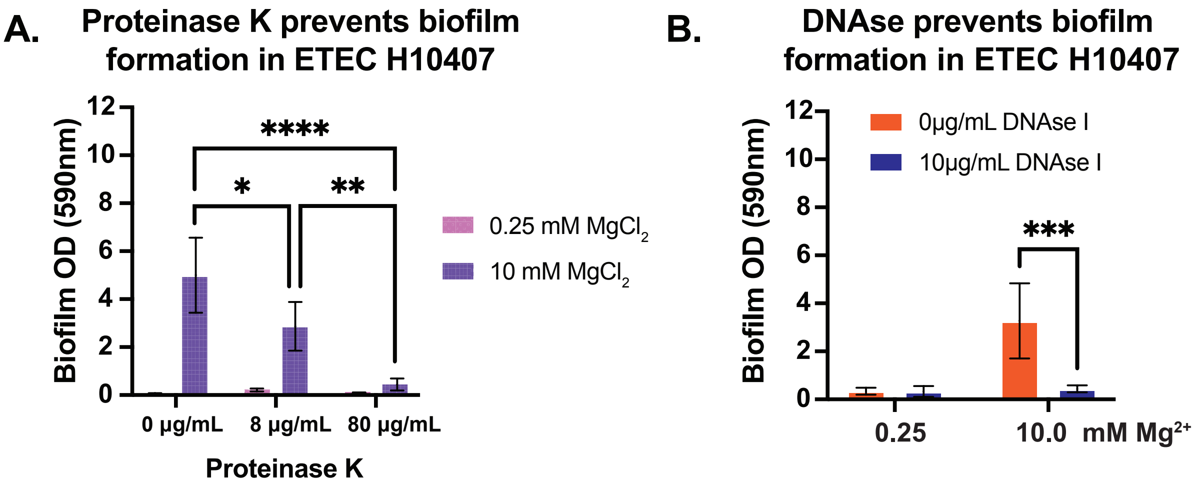

Fig. S3

Figure S3: ETEC biofilm formation is inhibited by proteinase K and DNase I. ETEC H10407 was grown in 4AA-lactate with 0.25 mM or 10 mM magnesium chloride and supplemented with or without proteinase K (8 μg/mL or 80 μg/mL) (A) or DNAse I (10 μg/mL) (B). Biofilms were analyzed via crystal violet staining. Data were analyzed via two-way ANOVA with Šídák's test for multiple comparisons; *, $p < 0.05$; **, $p < 0.01$, ****, $p < 0.0001$.

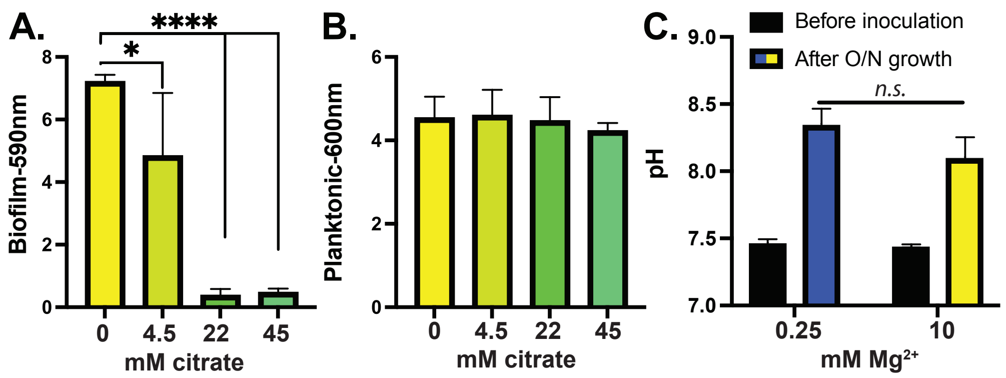

Fig. S4

Figure S4: Citrate inhibits biofilm formation and pH is unlikely to induce magnesium-dependent ETEC biofilms. ETEC H10407 wasgrown overnight in 4AA-lactate with 10 mM magnesium chloride and 0-, 4.5-, 22-, or 45 mM of sodium citrate then the liquid phase was separated from the culture tubes. Biofilm formation was measured by crystal violet staining (A) and the liquid phase cultures were measured by optical density (B). The pH of culture tubes containing 4AA-lactate culture media with 0.25 mM or 10 mM magnesium chloride was measured initially (black) and after overnight growth (blue or yellow) (C). Data are compiled from three replicates and were analyzed by one-way ANOVA with Dunnett's test for multiple comparisons (A, B) and Student's t-test (C); *, $p < 0.05$; ****, $p < 0.0001$.

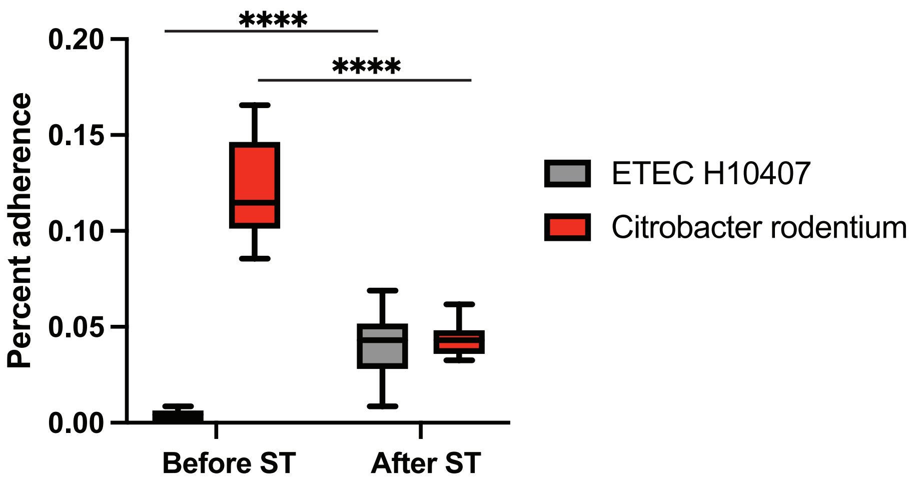

Fig. S5

Figure S5: ST intoxication promotes ETEC H10407 adherence at the expense of other enteric pathogens. Confluent T84 monolayers were treated with ST (100 ng) or left untreated for 1 hour before application of 104 CFUs of ETEC H10407 or C. rodentium 51459. The bacteria were allowed to adhere for 1 hour before non-adhered bacteria were removed by PBS washing three times. Adhered bacteria were enumerated by counting on LB-agar. Data are compiled from three replicates and analyzed by t-tests between untreated and ST-treated monolayers, ****, $p < 0.0001$. As shown, ST enhances the ability of ETEC H10407 to adhere to T84 monolayers but suppresses the ability of C. rodentium to adhere T84 monolayers.

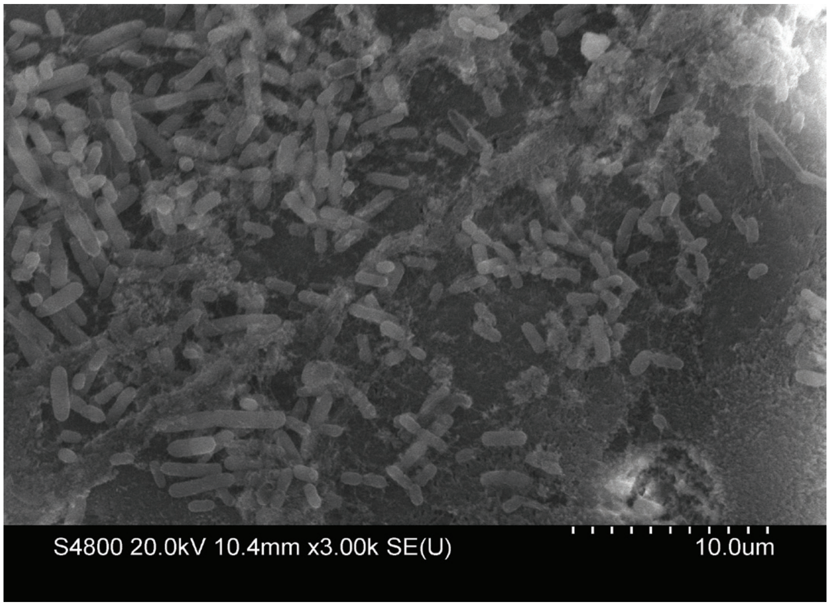

Fig. S6

Figure S6: Attachment of H10407 biofilm to a murine intestinal villus. Scanning electron micrograph of small intestinal sample from a neonatal mouse infected with 107 ETEC H10407 grown in 4AA-lactate with 10 mM magnesium chloride. Pictured are biofilm bacteria attached to a murine villus. Pictured at 3000× magnification, scale bar represents 10µm.

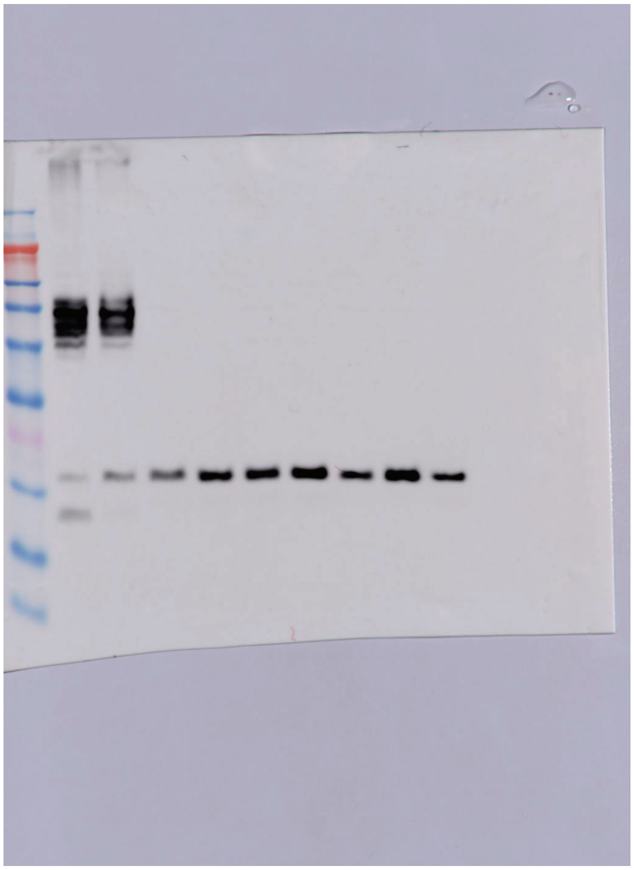

Uncropped image for Figure S1A

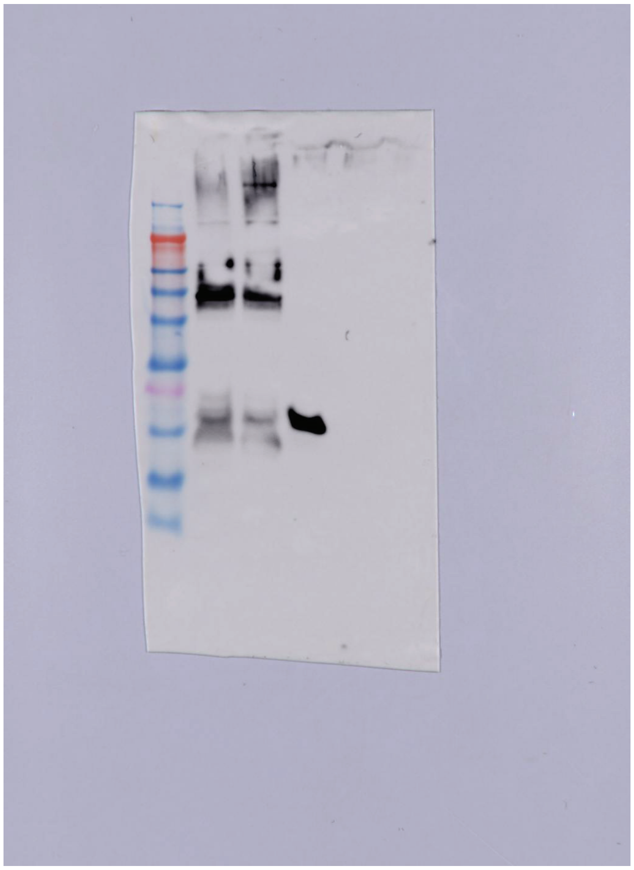

Uncropped image for Figure S1D
